# RE: Comment On: Association of HOTAIR rs1899663 G>T Polymorphism with Colorectal Cancer in the Turkish Population: A Case–Control Study

**DOI:** 10.5152/tjg.2023.231792

**Published:** 2023-07-01

**Authors:** Berrin Yalınbaş Kaya, Betül Peker, Fuzuli Tuğrul, Özge Alkan Tali, Süleyman Bayram

**Affiliations:** 1 Department of Internal Diseases and Gastroenterology, Eskişehir City Hospital, Eskişehir, Turkey; 2 Department of Pathology, Yunus Emre State Hospital, Eskişehir, Turkey; 3 Department of Radiation Oncology, Eskişehir City Hospital, Eskişehir, Turkey; 4 Department of Internal Diseases, Eskişehir City Hospital, Eskişehir, Turkey; 5 Department of Public Health Nursing, Adıyaman University Faculty of Health Sciences, Adıyaman, Turkey

Dear Editor,

First of all, we would like to thank the authors for their comments and analyses regarding the results of our article.^1^ As stated by the authors, these comments and analyses support and strengthen the results of our article.^1^

In similar genetic association studies, DNA samples are usually isolated from peripheral blood. However, the TaqMan allelic discrimination assay used in this study is a very sensitive genotyping method and does not work in any genetic changes that occur in the genotyped DNA sequence. To ensure quality control, genotyping was performed without knowledge of the subjects’ case/control status, and a 15% random sample of cases and controls was genotyped twice by different persons and reproducibility was 100%. Therefore, there cannot be any bias regarding genotyping.

As reported in our article^1^, the G allele of *HOTAIR* rs1899663 G>T polymorphism has been considered protective in some studies.^2^ But, there are studies in the literature reporting that the T allele of *HOTAIR* rs1899663 G>T polymorphism increases the risk of cancer.^3^ Contrary to these results, Hassanzarei et al^4^ reported that the T allele of rs1899663 G>T polymorphism of *HOTAIR* gene was protective against breast cancer in the Iranian population. The basis of *HOTAIR* rs1899663 G>T polymorphism is the replacement of guanine by thymine at the 12th chromosome at 53967210th position.^5^ *HOTAIR* rs1899663 G>T polymorphism is located in the intronic region of *HOTAIR* gene (in the second intron site in 4903 position).^5^ Many researchers have focused on finding the role of functional intronic polymorphisms because genetic polymorphisms in introns can affect alternative gene splicing and alter the expression of distant genes.^6^ In silico analysis via HaploReg v4 software shows that the *HOTAIR *rs1899663 G>T polymorphism has altered the binding affinity of transcription factors such as PAX-4, SOX, SPZ1, and ZFP281.^6^ However, there is no evidence in the literature regarding the clinical and biological significance of neither the G allele nor the T allele of *HOTAIR* rs1899663 G>T polymorphism. When the biological significance of the G and T alleles of *HOTAIR* rs1899663 G>T polymorphism is understood with further analysis (such as reporter gene assays), it will be better understood whether the G allele is protective or the T allele increases risk.
